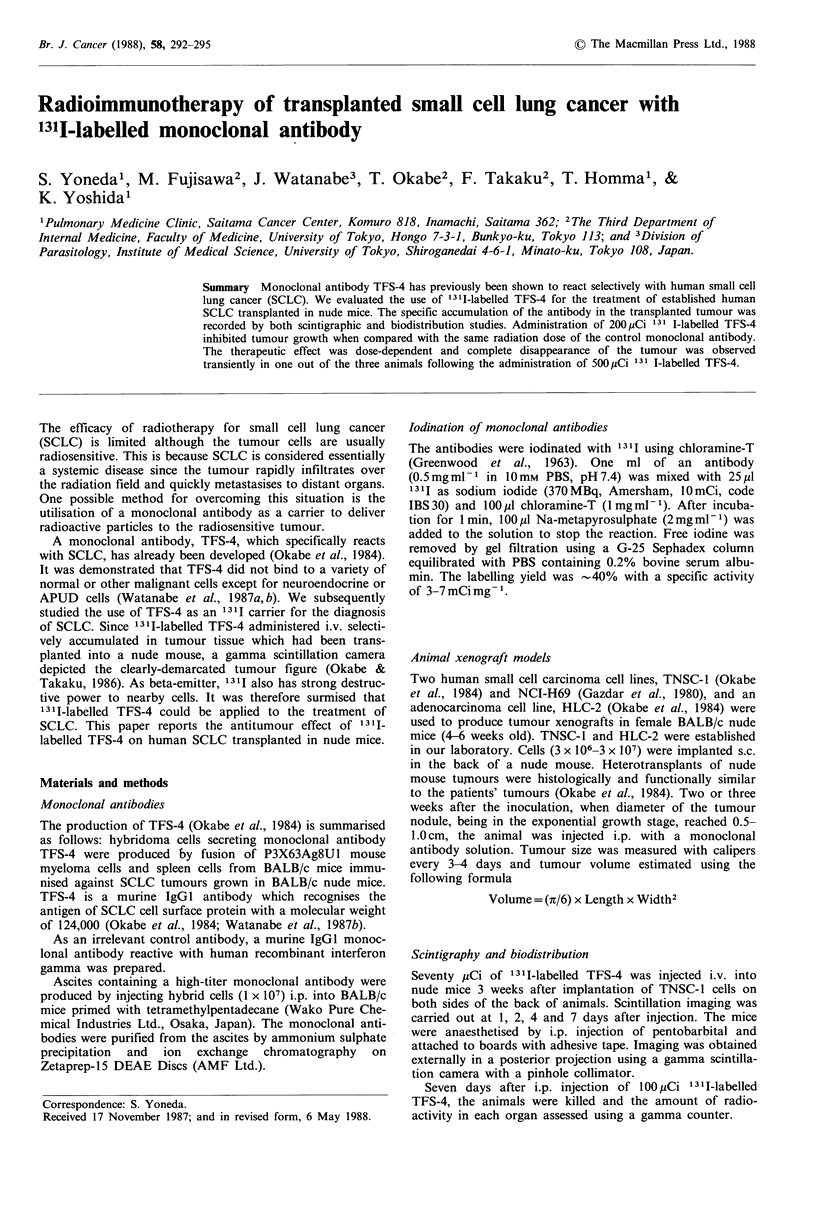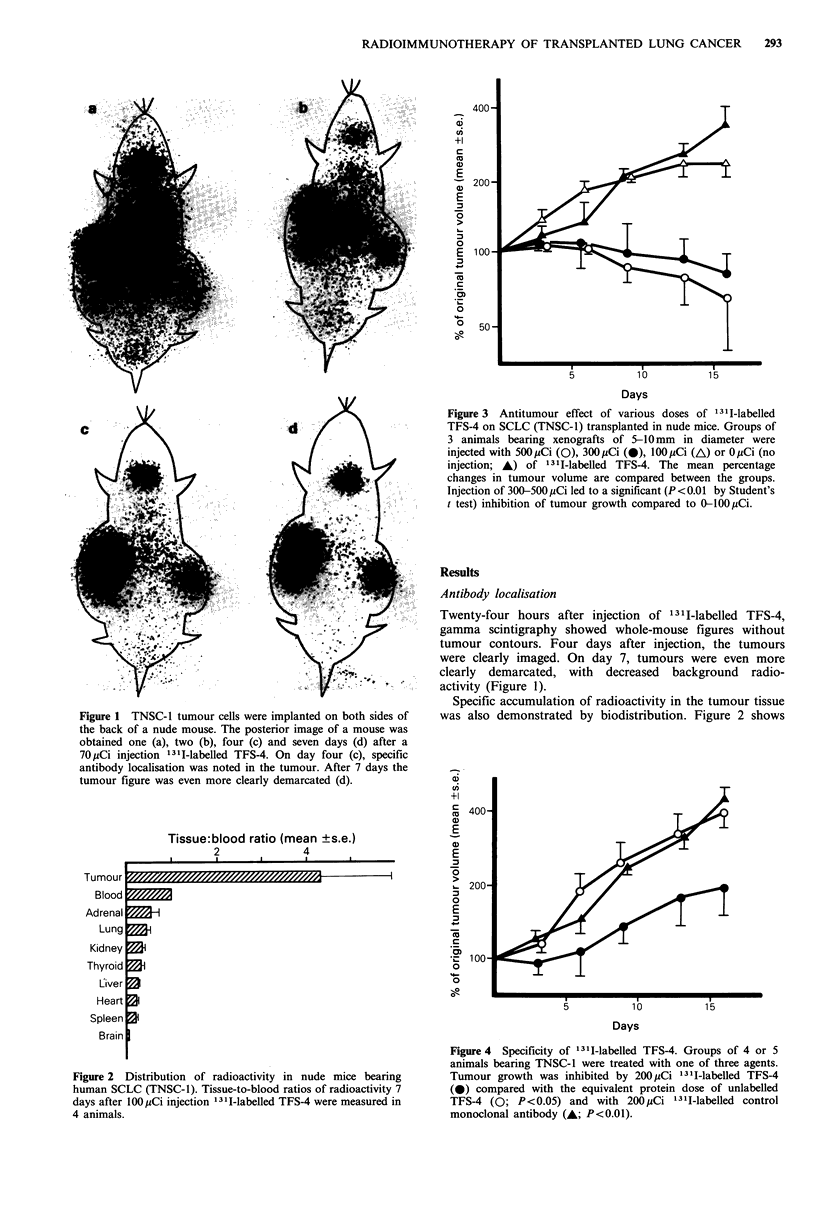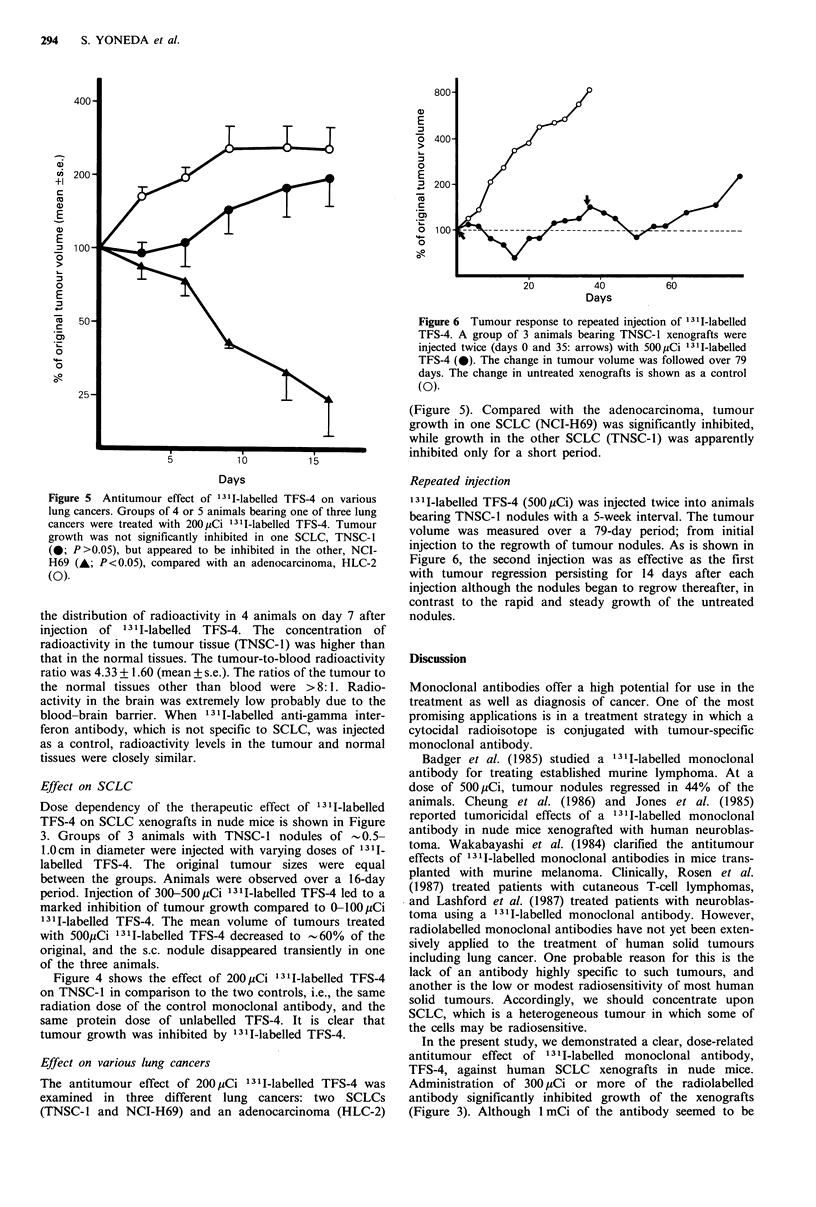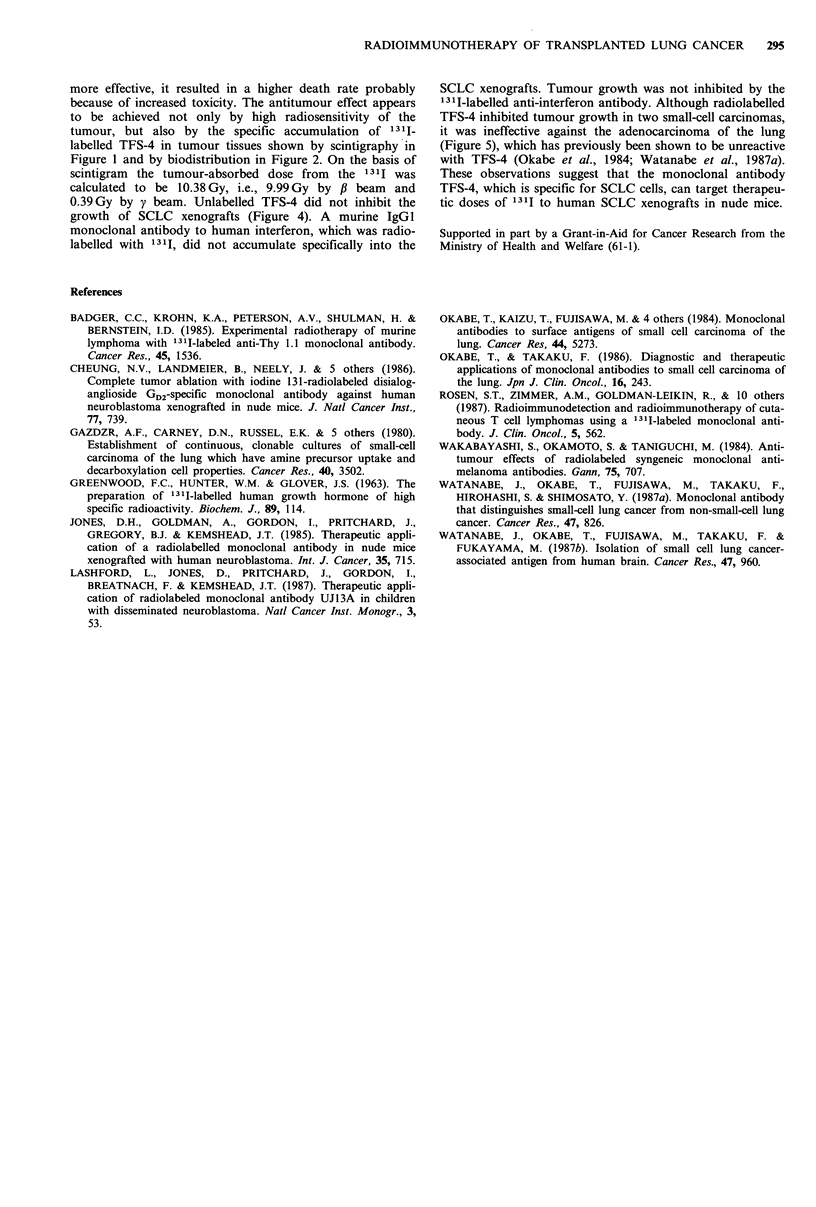# Radioimmunotherapy of transplanted small cell lung cancer with 131I-labelled monoclonal antibody.

**DOI:** 10.1038/bjc.1988.206

**Published:** 1988-09

**Authors:** S. Yoneda, M. Fujisawa, J. Watanabe, T. Okabe, F. Takaku, T. Homma, K. Yoshida

**Affiliations:** Pulmonary Medicine Clinic, Saitama Cancer Center, Japan.

## Abstract

**Images:**


*Br. J. Cancer* (1988), **58**, 292–295 © The Macmillan Press Ltd., 1988

### Radioimmunotherapy of transplanted small cell lung cancer with $^{131}$I-labelled monoclonal antibody

S. Yoneda[1], M. Fujisawa[2], J. Watanabe[3], T. Okabe[2], F. Takaku[2], T. Homma[1], & K. Yoshida[1]

[1]*Pulmonary Medicine Clinic, Saitama Cancer Center, Komuro 818, Inamachi, Saitama 362;* [2]*The Third Department of Internal Medicine, Faculty of Medicine, University of Tokyo, Hongo 7-3-1, Bunkyo-ku, Tokyo 113; and* [3]*Division of Parasitology, Institute of Medical Science, University of Tokyo, Shiroganedai 4-6-1, Minato-ku, Tokyo 108, Japan.*

**Summary** Monoclonal antibody TFS-4 has previously been shown to react selectively with human small cell lung cancer (SCLC). We evaluated the use of $^{131}$I-labelled TFS-4 for the treatment of established human SCLC transplanted in nude mice. The specific accumulation of the antibody in the transplanted tumour was recorded by both scintigraphic and biodistribution studies. Administration of 200 $\mu$Ci $^{131}$ I-labelled TFS-4 inhibited tumour growth when compared with the same radiation dose of the control monoclonal antibody. The therapeutic effect was dose-dependent and complete disappearance of the tumour was observed transiently in one out of the three animals following the administration of 500 $\mu$Ci $^{131}$ I-labelled TFS-4.

The efficacy of radiotherapy for small cell lung cancer (SCLC) is limited although the tumour cells are usually radiosensitive. This is because SCLC is considered essentially a systemic disease since the tumour rapidly infiltrates over the radiation field and quickly metastasises to distant organs. One possible method for overcoming this situation is the utilisation of a monoclonal antibody as a carrier to deliver radioactive particles to the radiosensitive tumour.

A monoclonal antibody, TFS-4, which specifically reacts with SCLC, has already been developed (Okabe *et al.*, 1984). It was demonstrated that TFS-4 did not bind to a variety of normal or other malignant cells except for neuroendocrine or APUD cells (Watanabe *et al.*, 1987*a*,*b*). We subsequently studied the use of TFS-4 as an $^{131}$I carrier for the diagnosis of SCLC. Since $^{131}$I-labelled TFS-4 administered i.v. selectively accumulated in tumour tissue which had been transplanted into a nude mouse, a gamma scintillation camera depicted the clearly-demarcated tumour figure (Okabe & Takaku, 1986). As beta-emitter, $^{131}$I also has strong destructive power to nearby cells. It was therefore surmised that $^{131}$I-labelled TFS-4 could be applied to the treatment of SCLC. This paper reports the antitumour effect of $^{131}$I-labelled TFS-4 on human SCLC transplanted in nude mice.

### Materials and methods

### *Monoclonal antibodies*

The production of TFS-4 (Okabe *et al.*, 1984) is summarised as follows: hybridoma cells secreting monoclonal antibody TFS-4 were produced by fusion of P3X63Ag8U1 mouse myeloma cells and spleen cells from BALB/c mice immunised against SCLC tumours grown in BALB/c nude mice. TFS-4 is a murine IgG1 antibody which recognises the antigen of SCLC cell surface protein with a molecular weight of 124,000 (Okabe *et al.*, 1984; Watanabe *et al.*, 1987*b*).

As an irrelevant control antibody, a murine IgG1 monoclonal antibody reactive with human recombinant interferon gamma was prepared.

Ascites containing a high-titer monoclonal antibody were produced by injecting hybrid cells ($1 \times 10^7$) i.p. into BALB/c mice primed with tetramethylpentadecane (Wako Pure Chemical Industries Ltd., Osaka, Japan). The monoclonal antibodies were purified from the ascites by ammonium sulphate precipitation and ion exchange chromatography on Zetaprep-15 DEAE Discs (AMF Ltd.).

Correspondence: S. Yoneda.
Received 17 November 1987; and in revised form, 6 May 1988.

### *Iodination of monoclonal antibodies*

The antibodies were iodinated with $^{131}$I using chloramine-T (Greenwood *et al.*, 1963). One ml of an antibody (0.5 mg ml$^{-1}$ in 10 mM PBS, pH 7.4) was mixed with 25 $\mu$l $^{131}$I as sodium iodide (370 MBq, Amersham, 10 mCi, code IBS 30) and 100 $\mu$l chloramine-T (1 mg ml$^{-1}$). After incubation for 1 min, 100 $\mu$l Na-metapyrosulphate (2 mg ml$^{-1}$) was added to the solution to stop the reaction. Free iodine was removed by gel filtration using a G-25 Sephadex column equilibrated with PBS containing 0.2% bovine serum albumin. The labelling yield was ~40% with a specific activity of 3–7 mCi mg$^{-1}$.

### *Animal xenograft models*

Two human small cell carcinoma cell lines, TNSC-1 (Okabe *et al.*, 1984) and NCI-H69 (Gazdar *et al.*, 1980), and an adenocarcinoma cell line, HLC-2 (Okabe *et al.*, 1984) were used to produce tumour xenografts in female BALB/c nude mice (4–6 weeks old). TNSC-1 and HLC-2 were established in our laboratory. Cells ($3 \times 10^6$–$3 \times 10^7$) were implanted s.c. in the back of a nude mouse. Heterotransplants of nude mouse tumours were histologically and functionally similar to the patients' tumours (Okabe *et al.*, 1984). Two or three weeks after the inoculation, when diameter of the tumour nodule, being in the exponential growth stage, reached 0.5–1.0 cm, the animal was injected i.p. with a monoclonal antibody solution. Tumour size was measured with calipers every 3–4 days and tumour volume estimated using the following formula

$$\text{Volume} = (\pi/6) \times \text{Length} \times \text{Width}^2$$

### *Scintigraphy and biodistribution*

Seventy $\mu$Ci of $^{131}$I-labelled TFS-4 was injected i.v. into nude mice 3 weeks after implantation of TNSC-1 cells on both sides of the back of animals. Scintillation imaging was carried out at 1, 2, 4 and 7 days after injection. The mice were anaesthetised by i.p. injection of pentobarbital and attached to boards with adhesive tape. Imaging was obtained externally in a posterior projection using a gamma scintillation camera with a pinhole collimator.

Seven days after i.p. injection of 100 $\mu$Ci $^{131}$I-labelled TFS-4, the animals were killed and the amount of radioactivity in each organ assessed using a gamma counter.

**Figure 1** TNSC-1 tumour cells were implanted on both sides of the back of a nude mouse. The posterior image of a mouse was obtained one (a), two (b), four (c) and seven days (d) after a 70 μCi injection $^{131}$I-labelled TFS-4. On day four (c), specific antibody localisation was noted in the tumour. After 7 days the tumour figure was even more clearly demarcated (d).

**Figure 2** Distribution of radioactivity in nude mice bearing human SCLC (TNSC-1). Tissue-to-blood ratios of radioactivity 7 days after 100 μCi injection $^{131}$I-labelled TFS-4 were measured in 4 animals.

**Figure 3** Antitumour effect of various doses of $^{131}$I-labelled TFS-4 on SCLC (TNSC-1) transplanted in nude mice. Groups of 3 animals bearing xenografts of 5–10 mm in diameter were injected with 500 μCi (○), 300 μCi (●), 100 μCi (△) or 0 μCi (no injection; ▲) of $^{131}$I-labelled TFS-4. The mean percentage changes in tumour volume are compared between the groups. Injection of 300–500 μCi led to a significant ($P<0.01$ by Student's $t$ test) inhibition of tumour growth compared to 0–100 μCi.

### Results

### *Antibody localisation*

Twenty-four hours after injection of $^{131}$I-labelled TFS-4, gamma scintigraphy showed whole-mouse figures without tumour contours. Four days after injection, the tumours were clearly imaged. On day 7, tumours were even more clearly demarcated, with decreased background radioactivity (Figure 1).

Specific accumulation of radioactivity in the tumour tissue was also demonstrated by biodistribution. Figure 2 shows

**Figure 4** Specificity of $^{131}$I-labelled TFS-4. Groups of 4 or 5 animals bearing TNSC-1 were treated with one of three agents. Tumour growth was inhibited by 200 μCi $^{131}$I-labelled TFS-4 (●) compared with the equivalent protein dose of unlabelled TFS-4 (○; $P<0.05$) and with 200 μCi $^{131}$I-labelled control monoclonal antibody (▲; $P<0.01$).

**Figure 5** Antitumour effect of $^{131}$I-labelled TFS-4 on various lung cancers. Groups of 4 or 5 animals bearing one of three lung cancers were treated with 200 μCi $^{131}$I-labelled TFS-4. Tumour growth was not significantly inhibited in one SCLC, TNSC-1 (●; $P>0.05$), but appeared to be inhibited in the other, NCI-H69 (▲; $P<0.05$), compared with an adenocarcinoma, HLC-2 (○).

the distribution of radioactivity in 4 animals on day 7 after injection of $^{131}$I-labelled TFS-4. The concentration of radioactivity in the tumour tissue (TNSC-1) was higher than that in the normal tissues. The tumour-to-blood radioactivity ratio was $4.33 \pm 1.60$ (mean ± s.e.). The ratios of the tumour to the normal tissues other than blood were >8:1. Radioactivity in the brain was extremely low probably due to the blood–brain barrier. When $^{131}$I-labelled anti-gamma interferon antibody, which is not specific to SCLC, was injected as a control, radioactivity levels in the tumour and normal tissues were closely similar.

*Effect on SCLC*

Dose dependency of the therapeutic effect of $^{131}$I-labelled TFS-4 on SCLC xenografts in nude mice is shown in Figure 3. Groups of 3 animals with TNSC-1 nodules of ~0.5–1.0 cm in diameter were injected with varying doses of $^{131}$I-labelled TFS-4. The original tumour sizes were equal between the groups. Animals were observed over a 16-day period. Injection of 300–500 μCi $^{131}$I-labelled TFS-4 led to a marked inhibition of tumour growth compared to 0–100 μCi $^{131}$I-labelled TFS-4. The mean volume of tumours treated with 500μCi $^{131}$I-labelled TFS-4 decreased to ~60% of the original, and the s.c. nodule disappeared transiently in one of the three animals.

Figure 4 shows the effect of 200 μCi $^{131}$I-labelled TFS-4 on TNSC-1 in comparison to the two controls, i.e., the same radiation dose of the control monoclonal antibody, and the same protein dose of unlabelled TFS-4. It is clear that tumour growth was inhibited by $^{131}$I-labelled TFS-4.

*Effect on various lung cancers*

The antitumour effect of 200 μCi $^{131}$I-labelled TFS-4 was examined in three different lung cancers: two SCLCs (TNSC-1 and NCI-H69) and an adenocarcinoma (HLC-2) (Figure 5). Compared with the adenocarcinoma, tumour growth in one SCLC (NCI-H69) was significantly inhibited, while growth in the other SCLC (TNSC-1) was apparently inhibited only for a short period.

**Figure 6** Tumour response to repeated injection of $^{131}$I-labelled TFS-4. A group of 3 animals bearing TNSC-1 xenografts were injected twice (days 0 and 35: arrows) with 500 μCi $^{131}$I-labelled TFS-4 (●). The change in tumour volume was followed over 79 days. The change in untreated xenografts is shown as a control (○).

*Repeated injection*

$^{131}$I-labelled TFS-4 (500 μCi) was injected twice into animals bearing TNSC-1 nodules with a 5-week interval. The tumour volume was measured over a 79-day period; from initial injection to the regrowth of tumour nodules. As is shown in Figure 6, the second injection was as effective as the first with tumour regression persisting for 14 days after each injection although the nodules began to regrow thereafter, in contrast to the rapid and steady growth of the untreated nodules.

### Discussion

Monoclonal antibodies offer a high potential for use in the treatment as well as diagnosis of cancer. One of the most promising applications is in a treatment strategy in which a cytocidal radioisotope is conjugated with tumour-specific monoclonal antibody.

Badger *et al.* (1985) studied a $^{131}$I-labelled monoclonal antibody for treating established murine lymphoma. At a dose of 500 μCi, tumour nodules regressed in 44% of the animals. Cheung *et al.* (1986) and Jones *et al.* (1985) reported tumoricidal effects of a $^{131}$I-labelled monoclonal antibody in nude mice xenografted with human neuroblastoma. Wakabayashi *et al.* (1984) clarified the antitumour effects of $^{131}$I-labelled monoclonal antibodies in mice transplanted with murine melanoma. Clinically, Rosen *et al.* (1987) treated patients with cutaneous T-cell lymphomas, and Lashford *et al.* (1987) treated patients with neuroblastoma using a $^{131}$I-labelled monoclonal antibody. However, radiolabelled monoclonal antibodies have not yet been extensively applied to the treatment of human solid tumours including lung cancer. One probable reason for this is the lack of an antibody highly specific to such tumours, and another is the low or modest radiosensitivity of most human solid tumours. Accordingly, we should concentrate upon SCLC, which is a heterogeneous tumour in which some of the cells may be radiosensitive.

In the present study, we demonstrated a clear, dose-related antitumour effect of $^{131}$I-labelled monoclonal antibody, TFS-4, against human SCLC xenografts in nude mice. Administration of 300 μCi or more of the radiolabelled antibody significantly inhibited growth of the xenografts (Figure 3). Although 1 mCi of the antibody seemed to be more effective, it resulted in a higher death rate probably because of increased toxicity. The antitumour effect appears to be achieved not only by high radiosensitivity of the tumour, but also by the specific accumulation of $^{131}$I-labelled TFS-4 in tumour tissues shown by scintigraphy in Figure 1 and by biodistribution in Figure 2. On the basis of scintigram the tumour-absorbed dose from the $^{131}$I was calculated to be 10.38 Gy, i.e., 9.99 Gy by $\beta$ beam and 0.39 Gy by $\gamma$ beam. Unlabelled TFS-4 did not inhibit the growth of SCLC xenografts (Figure 4). A murine IgG1 monoclonal antibody to human interferon, which was radiolabelled with $^{131}$I, did not accumulate specifically into the SCLC xenografts. Tumour growth was not inhibited by the $^{131}$I-labelled anti-interferon antibody. Although radiolabelled TFS-4 inhibited tumour growth in two small-cell carcinomas, it was ineffective against the adenocarcinoma of the lung (Figure 5), which has previously been shown to be unreactive with TFS-4 (Okabe *et al.*, 1984; Watanabe *et al.*, 1987*a*). These observations suggest that the monoclonal antibody TFS-4, which is specific for SCLC cells, can target therapeutic doses of $^{131}$I to human SCLC xenografts in nude mice.

Supported in part by a Grant-in-Aid for Cancer Research from the Ministry of Health and Welfare (61-1).

### References

BADGER, C.C., KROHN, K.A., PETERSON, A.V., SHULMAN, H. & BERNSTEIN, I.D. (1985). Experimental radiotherapy of murine lymphoma with $^{131}$I-labeled anti-Thy 1.1 monoclonal antibody. *Cancer Res.*, **45**, 1536.

CHEUNG, N.V., LANDMEIER, B., NEELY, J. & 5 others (1986). Complete tumor ablation with iodine 131-radiolabeled disialoganglioside $G_{D2}$-specific monoclonal antibody against human neuroblastoma xenografted in nude mice. *J. Natl Cancer Inst.*, **77**, 739.

GAZDZR, A.F., CARNEY, D.N., RUSSEL, E.K. & 5 others (1980). Establishment of continuous, clonable cultures of small-cell carcinoma of the lung which have amine precursor uptake and decarboxylation cell properties. *Cancer Res.*, **40**, 3502.

GREENWOOD, F.C., HUNTER, W.M. & GLOVER, J.S. (1963). The preparation of $^{131}$I-labelled human growth hormone of high specific radioactivity. *Biochem. J.*, **89**, 114.

JONES, D.H., GOLDMAN, A., GORDON, I., PRITCHARD, J., GREGORY, B.J. & KEMSHEAD, J.T. (1985). Therapeutic application of a radiolabelled monoclonal antibody in nude mice xenografted with human neuroblastoma. *Int. J. Cancer*, **35**, 715.

LASHFORD, L., JONES, D., PRITCHARD, J., GORDON, I., BREATNACH, F. & KEMSHEAD, J.T. (1987). Therapeutic application of radiolabeled monoclonal antibody UJ13A in children with disseminated neuroblastoma. *Natl Cancer Inst. Monogr.*, **3**, 53.

OKABE, T., KAIZU, T., FUJISAWA, M. & 4 others (1984). Monoclonal antibodies to surface antigens of small cell carcinoma of the lung. *Cancer Res*, **44**, 5273.

OKABE, T., & TAKAKU, F. (1986). Diagnostic and therapeutic applications of monoclonal antibodies to small cell carcinoma of the lung. *Jpn J. Clin. Oncol.*, **16**, 243.

ROSEN, S.T., ZIMMER, A.M., GOLDMAN-LEIKIN, R., & 10 others (1987). Radioimmunodetection and radioimmunotherapy of cutaneous T cell lymphomas using a $^{131}$I-labeled monoclonal antibody. *J. Clin. Oncol.*, **5**, 562.

WAKABAYASHI, S., OKAMOTO, S. & TANIGUCHI, M. (1984). Antitumour effects of radiolabeled syngeneic monoclonal antimelanoma antibodies. *Gann*, **75**, 707.

WATANABE, J., OKABE, T., FUJISAWA, M., TAKAKU, F., HIROHASHI, S. & SHIMOSATO, Y. (1987*a*). Monoclonal antibody that distinguishes small-cell lung cancer from non-small-cell lung cancer. *Cancer Res.*, **47**, 826.

WATANABE, J., OKABE, T., FUJISAWA, M., TAKAKU, F. & FUKAYAMA, M. (1987*b*). Isolation of small cell lung cancer-associated antigen from human brain. *Cancer Res.*, **47**, 960.